# Antimicrobial Resistance and Genetic Diversity of *Pseudomonas aeruginosa* Strains Isolated from Equine and Other Veterinary Samples

**DOI:** 10.3390/pathogens12010064

**Published:** 2022-12-30

**Authors:** Marine Pottier, Sophie Castagnet, François Gravey, Guillaume Leduc, Corinne Sévin, Sandrine Petry, Jean-Christophe Giard, Simon Le Hello, Albertine Léon

**Affiliations:** 1 Research Department, LABÉO, 14053 Caen, France; 2 Inserm UMR 1311, Dynamicure, Normandie University, UNICAEN, UNIROUEN, 14000 Caen, France; 3 CHU de Caen, Service de Microbiologie, Avenue de la Côte de Nacre, 14033 Caen, France; 4 Anses, Normandy Laboratory for Animal Health, Physiopathology and Epidemiology of Equine Diseases Unit, 14430 Goustranville, France; 5 CHU de Caen, Service d’Hygiène Hospitalière, Avenue de la Côte de Nacre, 14033 Caen, France

**Keywords:** *Pseudomonas aeruginosa*, antimicrobial susceptibility, whole-genome sequencing and typing, equine, resistome

## Abstract

*Pseudomonas aeruginosa* is one of the leading causes of healthcare-associated infections in humans. This bacterium is less represented in veterinary medicine, despite causing difficult-to-treat infections due to its capacity to acquire antimicrobial resistance, produce biofilms, and persist in the environment, along with its limited number of veterinary antibiotic therapies. Here, we explored susceptibility profiles to antibiotics and to didecyldimethylammonium chloride (DDAC), a quaternary ammonium widely used as a disinfectant, in 168 *P. aeruginosa* strains isolated from animals, mainly Equidae. A genomic study was performed on 41 of these strains to determine their serotype, sequence type (ST), relatedness, and resistome. Overall, 7.7% of animal strains were resistant to carbapenems, 10.1% presented a multidrug-resistant (MDR) profile, and 11.3% showed decreased susceptibility (DS) to DDAC. Genomic analyses revealed that the study population was diverse, and 4.9% were ST235, which is considered the most relevant human high-risk clone worldwide. This study found *P. aeruginosa* populations with carbapenem resistance, multidrug resistance, and DS to DDAC in equine and canine isolates. These strains, which are not susceptible to antibiotics used in veterinary and human medicine, warrant close the setting up of a clone monitoring, based on that already in place in human medicine, in a one-health approach.

## 1. Introduction

*Pseudomonas aeruginosa* (*P. aeruginosa*) is a Gram-negative bacterium commonly found in water and soil and is considered an opportunistic pathogen for humans, animals, and plants [1,2]. It can simply be carried in humans or can lead to various types of acute or chronic infection, typically nosocomial infections such as hospital-acquired pneumonia, bloodstream infections, and urinary tract infections, especially in urinary catheterization in humans and in immunocompromised individuals [3]. 

In veterinary medicine, even though *P. aeruginosa* naturally colonizes animal surface tissues [4], infection is relatively uncommon. However, it does cover a diverse spectrum, from otitis, ulcerative keratitis, urinary tract infections, and pyoderma in dogs and cats [2,5,6,7], to mastitis in dairy cows, sheep and goats [2,8], hemorrhagic pneumonia in mink, otitis in chinchillas, and necrotic upper and lower respiratory tract lesions in snakes [9]. *P. aeruginosa* infections in animals, and particularly dog otitis cases, occur following improperly administered antibiotics [4]. In 2021 in France, according to the Résapath data, *Pseudomonas* spp. represented 10% and 8% of antibiograms performed on dog and horse samples, respectively [10]. 

*P. aeruginosa* is reported as a rare pathogen in horses, but it can cause opportunistic infections of the reproductive tract, lower respiratory tract, eyes, skin, and guttural pouch, especially after antimicrobial therapy [2,11,12,13]. 

Worldwide, in the horse breeding industry, *P. aeruginosa* and *Klebsiella pneumoniae* (*K. pneumoniae*) infections are screened via surveillance for *Taylorella equigenitalis* [14], which is the causative agent of contagious equine metritis [15]. For these three bacteria, the severity of the infection is variable in the mare. *P. aeruginosa* can cause endometritis [16,17,18] and infertility [19,20]. In 13 studies performed between 1996 and 2021 in different countries, *P. aeruginosa* represented between 0 and 11.7% of bacterial species found from either a uterine swab or uterine lavage [20]. The mare can be either a simple carrier in the absence of clinical signs, or infected in acute or chronic forms [21], characterized by vulvar discharge ranging from slight to abundant. Stallion do not usually show clinical signs of infection. Carriers (mare and stallion) remain vectors of transmission, as the bacterial cells can be found on the upper surface of the clitoris, in the clitoral fossa and sinuses, on the penis and sheath or, less frequently, in the urethra and bladder. Semen may also be contaminated by colonization of the stallion’s sex glands [14]. Biofilm production, which can lead to persistent infections, is also involved in this type of infection in mares [21]. 

*P. aeruginosa* can be found in various environments and be carried in the intestinal tract of healthy animals, considered as reservoirs for the bacteria [2,22], due to its ubiquity and versatile metabolism [23,24]. The scale of *P. aeruginosa* dissemination and infection cases can be reduced by judicious use of antimicrobial therapy and by control of the human and animal hospital environment through prophylactic measures and cleaning, drying, disinfection, and sterilization processes.

One of the challenges posed by *P. aeruginosa* infections is that they are difficult to treat effectively. Indeed, in addition to a natural resistance to many usual classes of antibiotics, the bacterium can also acquire resistances that can lead to multi-resistant phenotypes and ultimately extensive resistance [25]. The natural resistance of *P. aeruginosa* is mostly explained by the low permeability of its outer membrane, expression of the chromosomic cephalosporinase AmpC, and constitutive expression of the efflux pump MexAB-OprM [26]. Intrinsic resistance, which is associated with other resistance mechanisms, such as gene acquisition or gene mutational processes that modify the expression and function of chromosomally encoded mechanisms, can lead to high-level resistance and even therapeutic deadlock [27,28,29]. Other factors that increase the difficulty of treating *P. aeruginosa* infections include its ability to adhere to tissues, intracellular accumulation, and biofilm formation [30]. Antimicrobial resistance in *P. aeruginosa* has reached such a point that the World Health Organization has listed carbapenem-resistant *P. aeruginosa* among the critical priority pathogens for new antibiotic research and development [31]. In veterinary medicine, despite a lower prevalence of multidrug resistance in bacteria than found in human medicine, the low number of antibiotic treatments authorized impacts the issue of antimicrobial resistance [2,32].

Due to cross-resistance and/or co-resistance mechanisms, the overuse and misuse of disinfectants has also led to decreased susceptibility to important antimicrobials, such as chlorhexidine, hypochlorite, and didecyldimethylammonium chloride (DDAC) [33,34,35,36,37]. Decreased susceptibility can occur via natural selection or by reinforcement of an acquired resistance mechanism that allows for adaptation to the new environment [38]. Both for veterinary medicine in general and livestock in particular, antiseptics and disinfectants are critical elements in infectious agent management, including zoonosis and antimicrobial-resistant micro-organisms [39]. In the European Union, there are almost 250 different chemical compounds that are used, either alone or in combination, in disinfectant products, including quaternary-ammonium-based disinfectants [40]. DDAC is a quaternary ammonium compound employed as a biocide in various applications for private and professional use, from food and agriculture to leisure and medical equipment [41]. In veterinary settings, DDAC is considered one of the most commonly used biocides in Europe [42], where it is used for its detergent and disinfectant actions on floors, walls, accessories, examination tables, medicated equipment, and noninvasive medical devices [43].

Here, we studied animal *P. aeruginosa* strains isolated from 1996 to 2020 to: (1) determine their antimicrobial resistance profile and susceptibility to DDAC detergent–disinfectant; and (2) describe the genetic diversity of circulating populations and their resistome.

## 2. Materials and Methods

### 2.1. P. aeruginosa Bacterial Strains

We used 4 reference strains with the available genomes: ATCC15442 and ATCC27853 were obtained from the American Type Culture Collection (ATCC), and PAO1 and PA14 from the Institut Pasteur collection (Paris, France). ATCC15442 is recommended for disinfectant susceptibility testing [44], ATCC27853 is the reference for *Pseudomonas* spp. antibiotic susceptibility testing [45], PAO1 is the reference genome for the *P. aeruginosa* species [23], and PA14 is a highly virulent strain that represents the most common *P. aeruginosa* clonal group worldwide [46].

**Figure 1:**
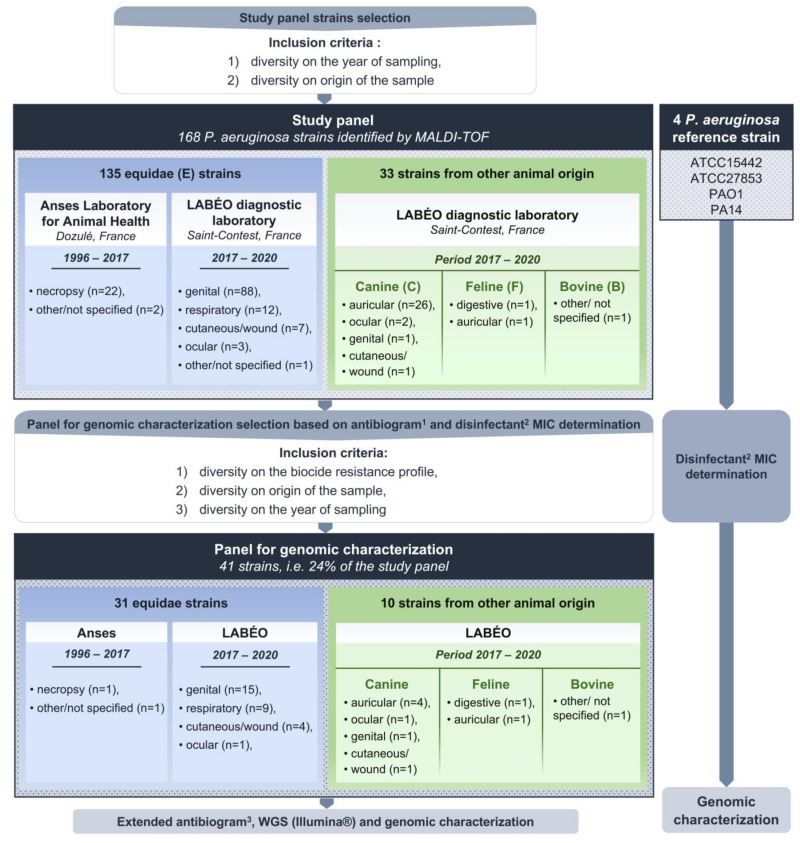
Selection diagram for the strains of the study. 1: six classes tested by disc diffusion method, i.e., 16 antibiotics for human and/or veterinary use according to the CASFM/EUCAST 2021 V1.0 list for *Pseudomonas* spp. and 4 antibiotics for veterinary use according to the CASFM 2013; 2: didecyldimethylammonium chloride tested by the broth dilution method; 3: in total 21 antibiotics for hospital or veterinary use. Anses: Agence nationale de sécurité sanitaire de l’alimentation, de l’environnement et du travail, B: bovine, C: canine, DDAC: didecyldimethylammonium chloride, E: equine, F: feline, MALDI-TOF: matrix-assisted laser desorption/ionization–time-of-flight, MIC: minimum inhibitory concentration, *P. aeruginosa*: *Pseudomonas aeruginosa*, WGS: whole-genome sequencing.

A further 168 *P. aeruginosa* strains isolated from animals (135 from equid origin, 30 from canine, 2 from feline, and 1 from bovine) were selected retrospectively (Figure 1 and Supplementary Table S1 Part 1): 24 were collected at the Anses, Normandy Laboratory for Animal Health (Goustranville, France), and the other 144 were isolated at the LABÉO diagnostic laboratory (Saint-Contest, France), from samples received for diagnostic (*ante* or *postmortem*) or screening analysis. Selected strains covered a diverse set in terms of the year of isolation and origin (animal species and type of sample). The Anses strains represented all isolated *P. aeruginosa* strains during the 1996–2017 period and included 22 from necropsies and two from other/unspecified sampling types. The LABÉO strains represented all veterinary antibiotic-resistant strains isolated and conserved during the 2017–2018 period (n = 27), and then all isolated *P. aeruginosa* strains during the 2019–2020 period (n = 117). The LABÉO’s strains were isolated from genital samples (n = 89), auricular samples (n = 27), respiratory samples (n = 12), cutaneous/wound samples (n = 8), ocular samples (n = 5), other/unspecified samples (n = 2), and digestive samples (n = 1). All strains were stored at −65 to −80 °C. Species identification was confirmed using matrix-assisted laser desorption/ionization-time-of-flight mass spectrometry (MALDI-TOF) (Microflex; Bruker Daltonik, Bremen, Germany). 

From these 168 strains, we selected 41 strains to constitute the panel for genomic characterization, including 31 equine strains, 7 canine strains, 2 feline strains, and 1 bovine-associated strain (Figure 1 and Table S1 Part 2). Selection was based on antibiotic susceptibility tests (AST), disinfectant minimum inhibitory concentration (MIC), and diversity in year of isolation and origin (animal species and type of sample) of the strains. Appendix A gives a summary of the temporal distribution and origin of the strains.

### 2.2. Antimicrobial Susceptibility Testing

#### 2.2.1. Antibiotic Susceptibility Testing

For the 168-strain study panel, ASTs were performed for 16 antipseudomonal antibiotics (Bio-Rad, Hercules, CA, USA), in association or not with a B-lactamase inhibitor: penicillin (piperacillin: PIL, piperacillin-tazobactam: PTZ, ticarcillin: TIC, ticarcillin-clavulanic acid: TCC), carbapenems (imipenem: IPM, meropenem: MEM), monobactams (aztreonam: ATM), cephalosporins (cefepime: FEP, ceftazidime: CZD, ceftolozane/tazobactam: CLT), phosphonic acid (fosfomycin: FOS), aminoglycosides (amikacin: AKN, gentamicin: GMN, tobramycin: TMN) and fluoroquinolones (ciprofloxacin: CIP, levofloxacin: LVX). The ASTs were performed on Mueller–Hinton agar (Becton Dickinson, Franklin Lakes, NJ) following the EUCAST guidelines for the *Pseudomonas* spp. disk diffusion method [45]. Other ASTs were performed for four veterinary antibiotics according to the NF U47-107 standard [47]: cephalosporins (cefquinome: CEQ, ceftiofur: XNL) and fluoroquinolones (enrofloxacin: ENR, marbofloxacin: MAR). For the 41 whole-genome-sequenced strains, ASTs were performed for the same panel and for one additional antibiotic: aminoglyco-side (netilmicin: NTM).

Antibiotic resistance breakpoints were obtained following the recommendations of EUCAST, CASFM, or veterinary CASFM, as summarized in Appendix B. For antibiotics with human medical uses (seven classes, all antibiotics tested except the veterinary-use antibiotics CEQ, XNL, ENR, and MAR), strains showing resistance in at least three antibiotic classes were considered multidrug-resistant (MDR). Strains with remaining susceptibility in one or two antibiotic categories were considered extensively drug-resistant (XDR) [48].

#### 2.2.2. Quaternary Ammonium Compound Susceptibility Testing

For the 168-strain study panel, the MIC of the detergent–disinfectant DDAC was assessed in triplicate per strain by the reference broth microdilution method [49] using cation-adjusted Mueller–Hinton Broth w/TES (Thermo Fisher Scientific, Waltham, MA, USA) at concentrations ranging from 0.5 to 1024 mg/L. Here, the threshold of decreased susceptibility (DS) was set at MIC > 62.9 mg/L, corresponding to the concentration of DDAC when combined with an alkylamine in a routine disinfectant diluted according to the manufacturer’s instructions for use.

### 2.3. Whole Genome Sequencing and Bioinformatics Analysis

Genomes of the reference strains ATCC27853 and PA14 were obtained from the European Nucleotide Archive (ENA) database with accession numbers CP015117 and ASWV01000001, respectively. The ATCC15442 and PAO1 sequences were obtained from GenBank with accession numbers GCF_000504485.1 and GCA_000006765.1, respectively.

The 41 strains were sequenced by the “Plateforme de Microbiologie Mutualisée P2M” (Institut Pasteur, Paris, France). A MagNA Pure 96 instrument (Roche Diagnostics, Meylan, France) was used for DNA extraction, a Nextera XT library kit (Illumina Inc., San Diego, CA, USA) was used for NGS library construction, and a NextSeq500 (Illumina Inc., San Diego, CA, USA) was used for sequencing. FastQC V0.11.0 [50] and MultiQC V1.9 [51] software were used for quality control checks on raw sequence data. The paired-end reads were preprocessed (filtered and trimmed) using fqCleaner [52,53,54,55,56,57,58,59], with a minimal read size of 70 bp and a Phred quality score of 28. De novo assembly was performed using SPAdes 3.12 [60] with a 50× minimum average sequencing depth, and Quast software V5.0 [61] was used for final assembly quality checks.

**Figure A1:**
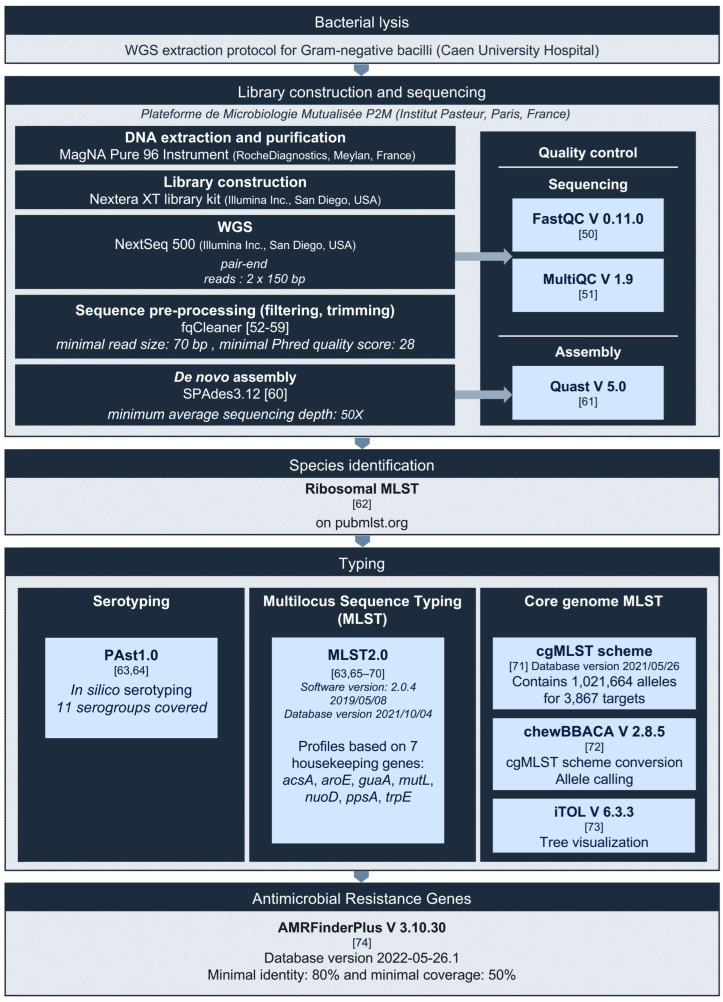
Bioinformatics pipeline for whole-genome sequencing and sequence analysis. [50,51,52,53,54,55,56,57,58,59,60,61,62,63,64,65,66,67,68,69,70,71,72,73,74]. cg: core genome; MLST: multilocus sequence typing; WGS: whole-genome sequencing.

Species identifications based on the sequences were validated with Ribosomal Multilocus Sequence Typing (rMLST), available at https://pubmlst.org/ (accessed on 25 November 2022) [62]. In silico, *P. aeruginosa* strain serotyping was performed using PAst1.0 software, available at https://cge.cbs.dtu.dk/services/PAst-1.0/ (accessed on 25 November 2022) [63,64]. Then, multilocus sequence typing (MLST) was performed on the sequence variation of 7 housekeeping genes using MLST2.0 software (version 2.0.4, 2019/05/08; database version 2021/10/04). This software uses the MLST allele sequence and profile obtained from PubMLST.org [63,65,66,67,68,69,70]. Finally, the core genome MLST was determined based on the *P. aeruginosa* MLST scheme, targeting 3867 loci (available on cgmlst.org at https://www.cgmlst.org/ncs/schema/16115339/locus/, accessed on 25 November 2022, database version 2021/05/26) [71]. Chewbacca software version 2.8.5 [72] was used for the cgMLST scheme conversion and allele calling. Finally, a neighbor-joining tree based on cgMLST was visualized with iTOL v6.3.3 [73]. The nucleotide sequences were submitted to AMRFinderPlus analysis (version 3.10.30, database version 2022-05-26.1) [74], with a minimal identity of 80% and minimal coverage of 50%, to identify antimicrobial resistance genes and known resistance-associated point mutations. The workflow steps from library construction and sequencing to sequence analysis were performed using the bioinformatics pipeline summarized in Appendix C (Figure A1). All 41 assembled genomes were deposited as BioProject PRJNA887012.

### 2.4. Statistical Analysis

All statistical tests were performed with GraphPad Prism version 9.0.0 for macOS (GraphPad Software, San Diego, CA, USA). 

Populations were tested for independence using Fisher’s exact test to determine whether DS to DDAC was linked to the nonsusceptibility of strains to antibiotics (MDR or XDR profiles). Then, to assess whether the distribution of the DS-to-DDAC phenotype was significant, Fisher’s tests were performed by comparing the numbers of each animal species to the numbers of the remaining population. The same method was applied to compare the distribution of the DS-to-DDAC phenotype by type of sample. For a given category (animal species and then type of sample), if the total number was less than five, it was not given a *p*-value as it was considered non-representative.

## 3. Results

### 3.1. Antimicrobial and DDAC Resistance Phenotypes

**Figure 2:**
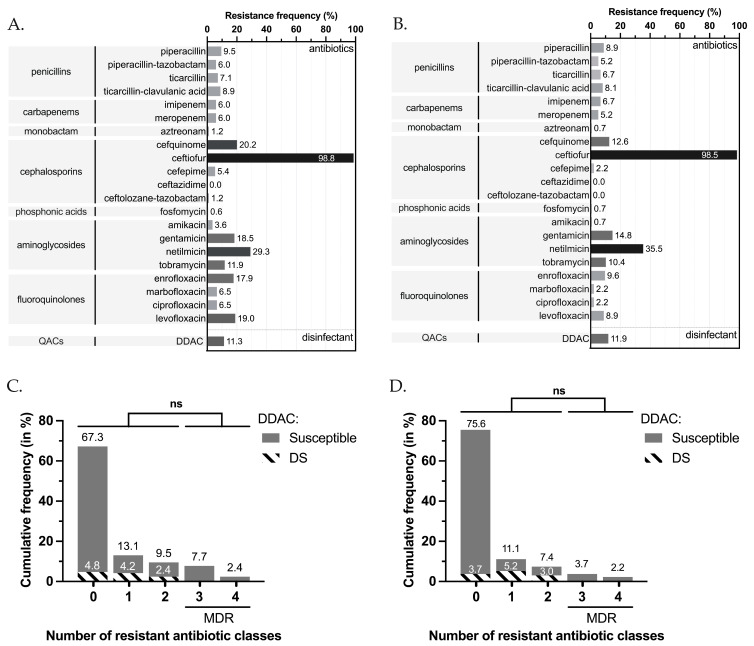
Antimicrobial resistance for veterinary strains. Frequency of resistance in *P. aeruginosa* strains tested for antibiotic susceptibility and antimicrobial resistance for the study panel (n = 168) (**A**) and for equine strains (n = 135) (**B**). Frequency histogram of the number of strains (in percent) showing at least one resistance for each different antibiotic class for the study panel (n = 168) (**C**) and for equine strains (n = 135) (**D**). Except for netilmicin, ceftiofur, cefquinome, marbofloxacin, and enrofloxacin, tested only for the panel for genomic characterization (n = 41), all other antimicrobials were tested for the study panel (n = 168). The four veterinary antibiotics (ceftiofur, cefquinome, marbofloxacin, and enrofloxacin) were excluded from the class-based resistance analysis for figure (**B**) Populations were tested for independence for DS to DDAC and loss of susceptibility to more than three categories of antibiotics using Fisher’s exact test. Ns: *p*-value > 0.05. DDAC: didecyldimethylammonium chloride; DS: decreased susceptibility; MDR: multidrug-resistant.

The highest rates of resistance were observed for the veterinary antibiotic XNL (98.8%), followed by NTM (29.3%), CEQ (20.2%), LVX (19.0%), GMN (18.5%), ENR (17.9%) and TMN (11.9%). Frequency of resistance was less than 10.0% for other antibiotics tested (Figure 2A). For equine strains, which represented a major part of our population, the rates were 98.5% for XNL, 35.5% for NTM, 14.8% for GMN, 12.6% for CEQ, and 10.4% for TMN (Figure 2B).

**Figure 3:**
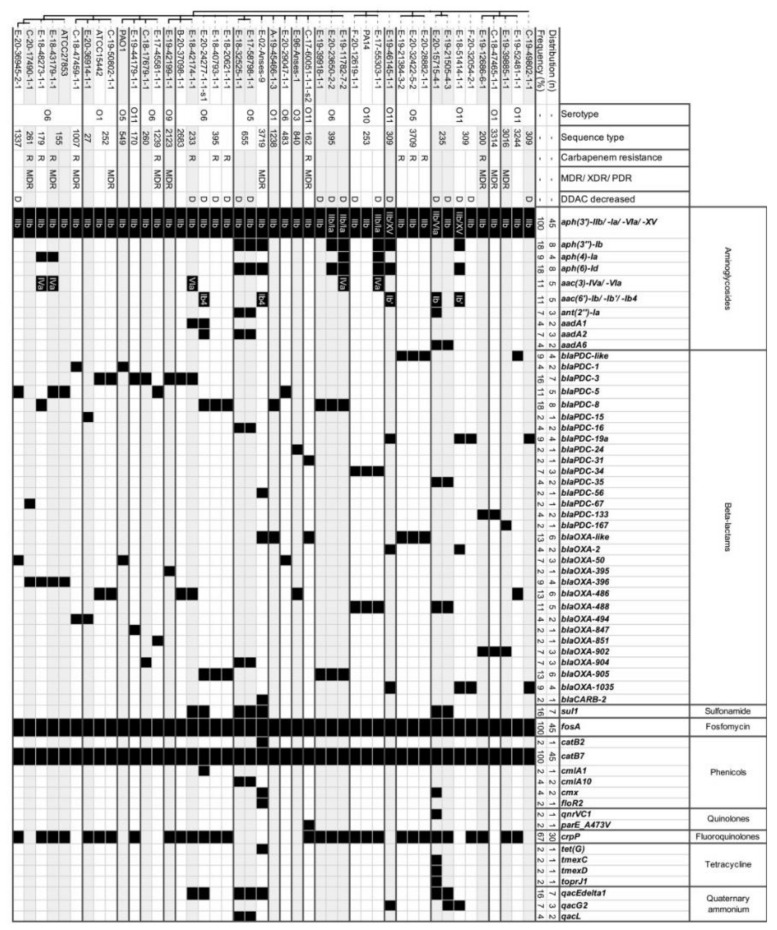
Antimicrobial-resistance-associated genes of strains from the panel for genomic characterization and for reference strains (n = 41 + 4). Strains were organized according to the cgMLST minimum distance tree. The figure lists serotype, sequence type, presence of a carbapenem resistance (imipenem and/or meropenem) phenotype, whether the strain was multidrug-resistant or not, and presence of decreased susceptibility to DDAC phenotype. The sub-variants of the *aph(3′)* gene were grouped, as well as the sub-variants of *aac(6′)*. Co-occurrences of these sub-variants are indicated and separated by a backslash. The “-like genes” were variants not referenced on AMRFinder that had less than 100% shared identity. C: canine, B: bovine, DDAC: didecyldimethylammonium chloride, E: equine, F: feline, MDR: multidrug-resistant, O: serotype, ST: sequence type.

Among the sequenced strains (n = 41), we found numerous genes conferring antibiotic resistance: some were constitutive for *P. aeruginosa*, and others were acquired. At least one resistance gene was associated with each main antibiotic class (Figure 3). A total of 11.9% (n = 20) of all veterinary strains were resistant to anti-*Pseudomonas* penicillins (at least one resistance among PIL, PTZ, TIC, TCC), 7.7% (n = 13) were resistant to carbapenems (IPM and/or MEM), 1.2% (n = 2) were resistant to monobactams (ATM), and 20.2% (n = 34) were resistant to cephalosporins (FEP, CZD, CLT, CEQ) (Figure 2A). For equine strains, 11.1% (n=15) were resistant to penicillins, 7.4% (n = 10) to carbapenems, and only one strain was resistant to monobactams (Figure 2B). For both classes of antibiotics, canine strains were the most resistant, with 16.7% (n = 5),10.0% (n = 3) and 3.3% (n = 1) of resistance, respectively (Figure 2B). These resistances were associated with the presence of resistance genes for β-lactams, including those coding CARB-2, the PSE family carbenicillin-hydrolyzing class A beta-lactamase (*bla_CARB-2_*), PDC, the cephalosporin-hydrolyzing class-C β-lactamase (*bla_PDC_* variants), and the OXA-family oxacillin-hydrolyzing class-D β-lactamases (*bla_OXA_*). The genes indicated as *bla_OXA_*-like and *bla_PDC_*-like were variants of *bla_OXA_* and *bla_PDC_*, respectively, that had less than 100% identity but were not known from the database. Note that no carbapenemase-coding genes were identified in the sequenced strains (Figure 3). For fosfomycin, only 0.6% (n = 1) of the veterinary population showed resistance, whereas the fosfomycin resistance glutathione transferase *(fosA*) coding gene was found in all sequenced strains. It was observed that 18.5% (n = 31) of the veterinary strains tested showed at least one resistance to aminoglycosides (AKN, GMN, NTM, TMN) (Figure 2A). Overall, 14.8% (n = 20) of equine strains were resistant to at least one aminoglycoside and 36.7% (n = 11) of canine strains (Figure 2B). Various antimicrobial resistance genes targeting this class of antibiotics were identified in the tested strains, including genes encoding aminoglycoside O-phosphotransferases (*aph* variants), N-acetyltransferases (*aac* variants), and aminoglycoside nucleotidyltransferases (*aad* variants) (Figure 3). For fluoroquinolones (CIP, LVX), at least one resistance was observed in 19.0% (n = 32) of veterinary strains. This rate was close (22.0%) when also considering veterinary antibiotics (ENR, MAR) (Figure 2A). The equine strains were resistant in 8.9% of cases (n = 12), although canine strains were resistant in 66.8% of cases (n = 20) (Figure 2B). For fluoroquinolone resistance, only gene-encoding protein CrpP was retrieved. For quinolone resistance, we identified genes encoding for the pentapeptide repeat protein QnrVC1 and modification of the amino acid sequence in the quinolone-resistance-determining region (QRDR) due to sequence alterations in position 473 of the *parE* gene. For sulfonamide, we found only the resistance gene encoding dihydropteroate synthase (*sul1*). For phenicols, we found type B chloramphenicol O-acetyltransferase (*catB* variants) and chloramphenicol efflux major facilitator superfamily (MFS) transporters (*cmlA*/*floR* variants and *cmx*). For tetracycline, genes coding the efflux MFS transporter Tet(G) and resistance-nodulation-division (RND) transporter efflux pump MexCD-OprJ were found. Interestingly, the *qacEdelta1*, *qacG2,* and *qacL* genes encoding small multidrug resistance (SMR) protein transporters were also found (Figure 3).

The study panel population showed various resistance profiles, with 67.3% (n = 113) of the strains displaying susceptibility to all 17 human antibiotics tested (seven classes), 13.1% (n = 22), and 9.5% (n = 16) showing nonsusceptibility to one and two antibiotic classes, respectively; 10.1% (n = 17) of strains were considered MDR (Figure 2B) but no strains were found to be XDR. The MDR profile concerned 30.0% (n = 9/30) of the canine-associated strains and 5.9% (n = 8/135) of the equine-associated strains, but was not associated with any other animal. Strain origins were auricular (n = 7/27; 25.9%), cutaneous/wound (n = 2/8; 25.0%), other/unspecified (n = 1/4; 25.0%), ocular (n = 1/5; 20.0%), respiratory (n = 1/12; 8.3%) and genital (n = 5/89; 5.6%). The feline- and bovine-associated strains did not present any resistance to antibiotics except the veterinary antibiotic ceftiofur. Note that the Anses strains were mostly susceptible to all the antibiotics tested, except for two strains, one of which was MDR.

Regarding DDAC, the MICs ranged from 8 to 128 mg/L, and decreased susceptibility (DS, MIC > 62.9 mg/L) was observed in 11.3% of tested strains (n = 19/168) (Figure 2C) and 11.9% of equine strains (n = 16/135) (Figure 2D). The DS to DDAC phenotypes was associated with patterns of antimicrobial drug resistance to up to two antibiotic classes: 4.8% showed no antibiotic resistance (3.7% for equine strains), 4.2% were resistant to one antibiotic class (5.2% for equine strains), and 2.4% were resistant to two antibiotic classes (3.0% for equine strains) (Figure 2C,D). The DS to DDAC phenotypes were observed since at least 2017 (from two in 2017 to seven in 2020; Table 1), largely in equine strains (n = 16) but also in feline (n = 2) and canine strains (n = 1) (Table 1). Before 2017, its presence cannot be evaluated, due to a lack of representativeness of the population. The DS to DDAC phenotype was significantly found in respiratory samples (n = 8, Fisher’s exact test, *p* < 0.0001), and it was also found in genital samples (n = 7); both were specifically associated with equines. The DS to DDAC phenotypes was also observed from ocular, cutaneous/wound, auricular, and digestive strains, but each only once (Table 1).

### 3.2. Genomic Diversity and Resistome Analysis

The 41 strains of the panel for genomic characterization were distributed into seven serotypes (Table 2). The three main serotypes were the same for all veterinary strains, and for the Equidae population in particular: serotypes O6 (39.0%), O11 (26.8%), and O5 (14.6%). Only O6 and O11, like serotypes O1 and O10, were found in more than one animal species. 

**Figure 4:**
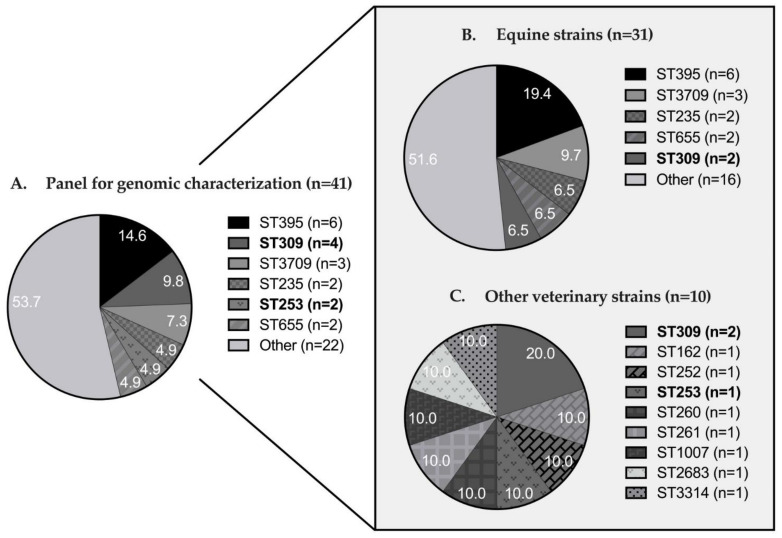
Multilocus sequence typing (MLST) of 41 sequenced strains. Representation of (**A**) the whole panel for genomic characterization, (**B**) equine strains, and (**C**) strains from other animal species (i.e., canine, feline, and bovine). The sequence types written in bold were found to be associated with several animal species. In figures (**A**,**B**), sequence types represented only once were grouped together. Figure (**A**) contained ST27, ST155, ST162, ST170, ST179, ST200, ST233, ST252, ST260, ST261, ST483, ST840, ST1007, ST1238, ST1239, ST1337, ST2123, ST2683, ST3016, ST3244, ST3314, and ST3719; figure (**B**) contained ST27, ST155, ST170, ST179, ST200, ST233, ST253, ST483, ST840, ST1238, ST1239, ST1337, ST2123, ST3016, ST3244, and ST3719. ST: Sequence type.

The 41 sequenced strains were distributed among 28 sequence types (ST) (Figure 4A): primarily ST395 (14.6%), ST309 (9.8%), ST3709 (7.3%), ST235 (4.9%), ST253 (4.9%), and ST655 (4.9%). The other 22 STs were represented once. A closer focus on the equine strains (Figure 4B) showed that the main STs were ST395 (19.4%), ST3709 (9.7%), and ST235-ST309-ST655 (6.5% for each), with the other 16 ST represented by one strain. In the other animal species, only ST309 was represented twice, and ST162, ST252, ST253, ST260, ST261, ST1007, ST2683, and ST3314 were represented once (Figure 4C). Only ST309 and ST253 were found in more than one animal species.

**Figure 5:**
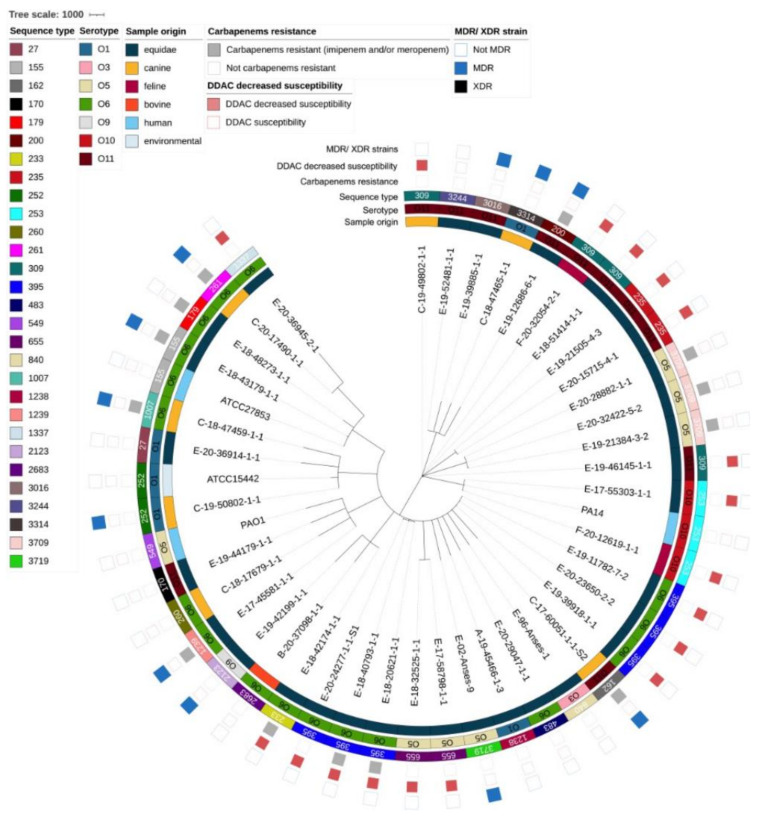
Minimum spanning tree of the strains from the panel for genomic characterization and reference strains (n = 41 + 4). Core genome MLST clustering according to the cgMLST *Pseudomonas aeruginosa* scheme previously published [71] and based on 3076 genes. The reference strains are ATCC15442, ATCC27853, PA14, and PAO1. The tree charts: origin of the strains (equine, canine, feline, bovine, human or environmental), serotype, sequence type, carbapenem resistance phenotype, DS to DDAC phenotype, and whether or not the strain was multidrug-resistant. C: canine, B: bovine, DDAC: didecyldimethylammonium chloride, E: equine, F: feline, MDR: multidrug-resistant, O: serotype, ST: sequence type, XDR: extensively drug-resistant.

This apparent diversity in serotypes and sequence types was confirmed by the cgMLST results charted in Figure 4. Of a total of 3867 loci searched, 3314 loci were present in the genome of all strains. Overall, strains diverged in distance from 14 to 3256 loci (average distance was 2893) and were well distributed according to origin (equine, canine, feline, bovine, human or environmental), year of collection, serotype, sequence type, carbapenem-resistance phenotype (major antibiotics reserved for human medicine), DS to DDAC phenotype, and whether or not the strain was multidrug-resistant (Figure 5).

None of the 41 sequenced strains showed a high level of antibiotic resistance (carbapenem resistance and MDR status) associated with DS to DDAC. However, three equine strains (E-18-20621-1-1, E-18-40793-1-1, and E-18-42174-1-1) showed a DS to DDAC associated with resistance to carbapenems. These strains were serotype O6: two were ST395, and one was ST233. The other equine strains presented either a DS-to-DDAC profile (n = 13), an MDR profile associated or not with resistance to carbapenems (n = 3 and n = 5, respectively), carbapenem resistance only (n = 4), or none of these phenotypes (n = 107). MDR status was assigned to 5 out of 7 of tested canine strains: three had resistance to carbapenems, and only one had DS to DDAC (C-19-49802-1-1). These strains were not associated with a single serotype or ST. The two feline strains, F-20-32054-2-1 (O11 and ST309) and F-20-12619-1-1 (O10 and ST253) did not show resistance to carbapenems or an MDR profile but had DS to DDAC. The bovine strain B-20-37098 (O6 and ST2683) was susceptible to antibiotics and DDAC.

## 4. Discussion

In France, since the 1990s, the surveillance of antimicrobial resistance in animals has been performed by the RESAPATH network [https://resapath.anses.fr/ accessed on 25 November 2022]. In 2020, the RESAPATH collected results from 71 member laboratories of 51,736 antibiograms performed on strains from dogs (27.3%), cattle (19.7%), poultry (19.7%), cats (10.8%), horses (7.4%) and pigs (7%) [75]. The Anses–Normandy Laboratory for Animal Health and LABÉO are both members of RESAPATH, with LABÉO providing the French network with the majority of horse antibiograms. In the 2006–2021 period, LABÉO performed 35,686 antibiograms on equine samples, and *P. aeruginosa* was the fourth most important pathogen isolated after group C streptococci, *Escherichia coli,* and *Staphylococcus aureus*. *P. aeruginosa* represented 3.6% (1367/37,686) of total isolated bacteria [76,77]. The majority of *P. aeruginosa* strains were isolated from respiratory tract samples (36.2%; 495/1367), followed by genital swabs (35.7%; 488/1367) and cutaneous samples (17.1%; 234/1367) [76,77]. 

This study set out to review the level of resistance of veterinary strains to the main human and veterinary antibiotics and to a common disinfectant, and to highlight the lack of anti-*Pseudomonas* therapies available in the veterinary field. It also enabled us to investigate and report the genomic diversity of these populations and the different antimicrobial resistance genes represented in them, and argue for the need to jointly study the Human–Animal–Environmental reservoirs. We selected 111/263 (42.0%) of the strains isolated from 2017 to 2020 at LABÉO to ensure a diversity of sample years and origins. This panel was completed by 24 equine strains provided by Anses and isolated during necropsies performed in the 1996–2017 period. Several animal species were included in this study; however, the small number of feline and bovine strains ruled out including these strains in a cross-species comparison. However, these feline and bovine strains did make it possible to determine whether some phenotypes are specific to one of the species.

For ASTs, the only antibiotics cited for testing by the veterinary CASFM 2021 [78] are gentamicin, amikacin, and ciprofloxacin. The observed resistance rates in our population were 18.5% for gentamicin and less than 10% for amikacin and ciprofloxacin. However, among the list of anti-pseudomonas antibiotics tested in this study, only ceftiofur, cefquinome, gentamicin, marbofloxacin, and enrofloxacin are currently marketed for veterinary use according to the Index of Veterinary Medicines authorized in France by the Anses [79]. Polymyxins can also be used but were not tested here. In this population, resistance rates for these antibiotics are above 20% for cephalosporins (up to 98.8% for ceftiofur) but less than 20% for fluoroquinolones. 

We expected to find this high rate (up to 98.8%) of resistance to ceftiofur [80,81,82], but as this antibiotic is taken into consideration for the choice of therapy by some diagnostic laboratories, we wanted to provide additional evidence of the low activity of ceftiofur on *P. aeruginosa*. For gentamicin and fluoroquinolones, the rates obtained were lower than those found by van Spijk et al. [83] between 2012 and 2015 on an equine hospital population. Note that for some of these antibiotics, there is no marketed medicine suitable for use in every animal species. Consequently, there is a significant lack of antibiotic-based solutions against *P. aeruginosa* in veterinary medicine. For all classes of antibiotics tested, canine strains had higher resistance rates than equine strains. This was particularly marked for fluoroquinolones (57.9% difference) and aminoglycosides (21.9% difference). The resistance values obtained here can be compared with a previous study performed at the nearby Caen University Hospital on strains isolated from patients between 2011 and 2020 [84]. The resistance rates found in *P. aeruginosa* strains were much lower than those obtained in human medicine for phosphonic acid (−32.7% for the veterinary population) and penicillins (−15.2%), and measurably lower for monobactams (−5.2%), cephalosporins (−4.8%) and carbapenems (−1.8%), but higher for fluoroquinolones (+3.4%) and aminoglycosides (+7.2%). It is surprising to note such a small difference in resistance rates in respect of carbapenems between veterinary and human populations, especially as carbapenems are not authorized and used in veterinary medicine in Europe, except in exceptional cases in university clinics. As found in other studies, the presence of a carbapenem resistance phenotype in animals is rarely associated with the presence of a carbapenemase. The presumed mechanisms would be the decreased permeability by deficiency of the outer membrane protein OprD2 [85], hyperproduction of the chromosomal cephalosporinase AmpC [86,87,88], or preferential overexpression of efflux pumps [89]. However, some rare cases of carbapenemase expression, especially VIM-2, have been reported in different countries in *P. aeruginosa* strains isolated from dogs, cattle, and fowl [90,91], but to our knowledge not yet in horses. Some studies even suggest the existence of zoonotic transmission from animals to humans [92] and from humans to animals [93,94], but in an anecdotal manner, including the case of a transmission of a VIM-2 strain from humans to animals in Brazil [95].

No pandrug-resistant or extensively drug-resistant strains were found, but 10.1% of the strains studied were categorized as MDR. Note that MDR strains were found in the more recent strains isolated at LABÉO, suggesting that equine *P. aeruginosa* strains have adapted to 3–4 classes of antibiotics. This finding corroborates similar previous studies [89] showing the existence of MDR strains in the veterinary population and extends this problem to Equidae. In comparison, in human medicine, 12.6% of *P. aeruginosa* strains analyzed have either MDR (11.9%) or XDR (0.7%) profiles [84].

From a genomic point of view, the 41 veterinary strains were quite diverse, which is consistent with the strain sampling system. These strains came from different types of environments depending on animal species and geographical location. In total, 28 different STs were identified. Note that among these STs, ST395 (n = 6), ST235 (n = 2), ST253 (n = 2), ST233 (n = 1), and ST27 (n = 1) were also found in the hospital isolates in a previous human study (patient only) [84]. Thus, 29.3% of the animal strains sequenced in this study shared an ST identified in patients at the University Hospital of Caen. ST235, representing 4.9% of the sequenced strains, was even considered in 2020 as among the top 10 *P. aeruginosa* high-risk clones worldwide based on prevalence, global spread, and association with MDR/XDR profiles and the extended-spectrum β-lactamases and carbapenemases [96]. However, in our study, ST235 strains were neither XDR, MDR, nor carbapenem-resistant, but only associated with a DS-to-DDAC phenotype. ST235 had already been found in Japan in dogs and cats at a rate of up to 21.1% [94]. Four ST309 strains were also identified in this study and were found in the human hospital environment [84]. It would be informative to study the genetic distance between veterinary and hospital strains in more detail, in particular using a cgMLST approach.

In terms of cgMLST, note that strains isolated from different animal species could have greater genomic proximity than strains isolated from the same animal species, which would be quite diversified. However, the data collected on our samples do not allow us to distinguish between cases of infection or colonization in the animal. This factor would determine whether or not strains from colonization and/or infection would be grouped on the phylogenetic tree. It would be also interesting to determine how close the human and animal strains were in order to get a picture of their potential capacity for human–animal transmission.

Concerning the resistome of the strains, some genes were only found to be associated with one animal species or within one ST. Except for *aph(3′)-IIb*, which is systematically present in *P. aeruginosa*, all the aminoglycoside resistance genes were specific to equine strains, as was also the case for some oxacillinases and the *bla_CARB-2_* gene and for the genes for resistance to sulfonamides, phenicols (except *catB7*), tetracycline, the *qnrVC1* gene targeting quinolones, and resistance genes to quaternary ammonium. The resistome associated with the other species was thus less diverse. On the whole, for the same ST, the strains had similar, but not always identical, resistomes. In ST252, ST655, and ST3709, only one resistome was found by ST, whereas for ST155, ST235, ST253, ST309, and ST395, various antimicrobial resistance gene profiles were found in each, although similar. Moreover, the same resistome could lead to various resistance profiles. For example, the canine-associated MDR strain C-19-50802-1-1 presented the same resistome as the non-MDR reference strain ATCC15442, implying potentially mutational mechanisms conferring higher resistance to the canine-associated strain.

This study also determined the MIC of *P. aeruginosa* strains to a quaternary ammonium compound, DDAC, that is widely used in veterinary hospital disinfectants and as a biocide in various applications. Considering a MIC > 62.9 mg/L (corresponding to the concentration of DDAC in the commercial disinfectant solution), there was DS to DDAC in 11.0% of our strains and this DS was not associated with MDR *P. aeruginosa* profiles. MICs of DDAC greater than or equal to our DS threshold have also been shown for veterinary strains isolated between 1994 and 2003 in the USA, but on a smaller scale (2.9% of 175 *P. aeruginosa* strains) [81]. In contrast, at Caen University Hospital, this profile was found for 38.9% of strains, spanning both human strains and hospital-environment strains, that were significantly more associated with MDR and XDR *P. aeruginosa* profiles and more prevalent in the hospital environment (62.5% of them) than in human strains (28.2%) [84]. In our population, the fact that we included strains potentially from individuals and non-hospital veterinary environments likely leads to a lower calculated rate of resistance. These strains must have been less frequently exposed to DDAC than strains from the hospital environment, which is obviously regularly disinfected. The underlying molecular mechanisms of this phenotype are not yet fully elucidated, but the initial evidence points to MexAB-OprM pump efflux overexpression [84]. 

## 5. Conclusions

To the best of our knowledge, this is the biggest and most representative phenotypic and genomic study on *P. aeruginosa* strains isolated from Equidae. With the implementation of whole-genome sequencing and genomic approaches, we were able to assess the diversity and the resistome of the different strains. Such a strategy is destined to become an indispensable tool for monitoring infections and the dissemination of resistance. In contrast to hospital-acquired human infections, our results point to a high diversity of *P. aeruginosa* populations as causative agents in equine infections. Because more than 10% of animal strains showed an MDR phenotype, horses may be considered reservoirs of antimicrobial resistance in *P. aeruginosa*. Moreover, a significant number of isolates were resistant to carbapenems (7.7%), which are antimicrobials non-authorized in veterinary medicine. This highlights the need for a global approach in epidemiological studies. Our data also pointed out that attention should be paid to the use of disinfectants such as DDAC, constituting a selective pressure for the persistence of less susceptible strains. Our robust data are the foundation for further monitoring *P. aeruginosa* resistant strains and optimizing antimicrobial therapies in veterinary medicine via a one-health approach.

## Figures and Tables

**Table 1:** Distribution of the decreased-susceptibility-to-DDAC phenotype in the study panel (n = 168) according to the year of strain isolation, animal species, and sample type. *p*-values of Fisher’s test of independence comparing the category against the rest of the population.

Year of Sampling	DDAC Status		
	Susceptible	DS	
	n *(%)*		
1996	1 (100.0)	-	
1997	1 (100.0)	-	
1998	2 (100.0)	-	
1999	2 (100.0)	-	
2002	2 (100.0)	-	
2003	2 (100.0)	-	
2004	3 (100.0)	-	
2005	3 (100.0)	-	
2007	1 (100.0)	-	
2008	1 (100.0)	-	
2010	3 (100.0)	-	
2011	1 (100.0)	-	
2015	1 (100.0)	-	
2017	7 (77.8)	2 (22.2)	
2018	14 (73.7)	5 (26.3)	
2019	61 (92.4)	5 (7.6)	
2020	44 (86.3)	7 (13.7)	
**Total**	**149 (88.7)**	**19 (11.3)**	
**Sample Origin**	**DDAC Status**		***p-values***
	**Susceptible**	**DS**	
	n ***(%)***		
Equine	119 (88.1)	16 (11.9)	>0.9999
Canine	29 (96.7)	1 (3.3)	0.2023
Feline	-	2 (100)	-
Bovine	1 (100)	-	-
**Total**	**149 (88.7)**	**19 (11.3)**	**-**
**Type of Sample**	**DDAC status**		***p-values***
	**Susceptible**	**DS**	
	n ***(%)***		
**Genital**	**82 (92.1)**	**7 (7.9)**	**0.1504**
Other animal species	1 (100)	-	-
Equine	81 (92)	7 (8)	-
**Auricular**	**26 (96.3)**	**1 (3.7)**	**0.3164**
Other animal species	26 (96.3)	1 (3.7)	-
**Necropsy**	**22 (100)**	**-**	**0.139**
Equine	22 (100)	-	-
**Respiratory**	**4 (33.3)**	**8 (66.7)**	**<0.0001**
Equine	4 (33.3)	8 (66.7)	-
**Cutaneous/wound**	**7 (87.5)**	**1 (12.5)**	**>0.9999**
Other animal species	1 (100)	-	-
Equine	6 (85.7)	1 (14.3)	-
**Ocular**	**4 (80)**	**1 (20)**	**0.4555**
Other animal species	1 (50)	1 (50)	-
Equine	3 (100)	-	-
**Other/not specified**	**4 (100)**	**-**	**-**
Other animal species	1 (100)	-	-
Equine	3 (100)	-	-
**Digestive**	**-**	**1 (100)**	**-**
Other animal species	-	1 (100)	-
**Total**	**149 (88.7)**	**19 (11.3)**	**-**

DDAC: didecyldimethylammonium chloride; DS: decreased susceptibility.

**Table 2:** *P. aeruginosa* serotype distribution (n = 41, panel for genomic characterization).

Strain Origin	Serotype							
	O1	O3	O5	O6	O9	O10	O11	Total
Equine	2	1	6	12	1	1	8	31
Canine	2			3			2	7
Feline						1	1	2
Bovine				1				1
Total n (%)	4 (9.8)	1 (2.4)	6 (14.6)	16 (39.0)	1 (2.4)	2 (4.9)	11 (26.8)	41 (100.0)

O: serotype.

## Data Availability

The datasets generated and analyzed during the current study are available via https://doi.org/10.6084/m9.figshare.c.6350123 accessed on 25 November 2022. All the DNA sequences of the present study can be downloaded in the BioProject PRJNA887012.

## Supplementary Materials

The following supporting information can be downloaded at: https://www.mdpi.com/article/10.3390/pathogens12010064/s1, Table S1: Characteristics of the strains used in the study (Part 1) and for genomic characterization (Part 2). Table S2: Excel file of antimicrobial resistance-associated genes of strains from the panel for genomic characterization and for reference strains (n = 41 + 4).

## Appendix A

**Table A1:** Distribution of the decreased susceptibility-to-DDAC phenotype in the panel for genomic characterization (n = 41), according to the year of strain isolation, animal species and sampling.

Year of Sampling	DDAC Status		Total n
	Susceptible n	DS n	
1996	1	-	1
2002	1	-	1
2017	2	2	4
2018	5	5	10
2019	8	5	13
2020	6	6	12
**Total**	**23**	**18**	**41**
**Sample origin**	**DDAC status**		**Total** n
	**Susceptible** n	**DS** n	
Equine	16	15	31
Canine	6	1	7
Feline	-	2	2
Bovine	1	-	1
**Total**	**23**	**18**	**41**
**Type of sample**	**DDAC status**		**Total** n
	**Susceptible** n	**DS** n	
Genital	10	6	16
Respiratory	1	8	9
Auricular	4	1	5
Cutaneous/wound	4	1	5
Other/not specified	2	-	2
Ocular	1	1	2
Digestive	-	1	1
Necropsy	1	-	1
**Total**	**23**	**18**	**41**

DDAC: didecyldimethylammonium chloride; DS: decreased susceptibility.

## Appendix B

**Table A2:** Zone diameter breakpoints used for clinical interpretation of inhibition zone diameters for all *P. aeruginosa* strains.

	Antibiotics	Used for MDR/XDR Categorization	Source	Zone Diameter Breakpoints (mm)		
				S≥	R<	ATU
Penicillins	Piperacillin	yes	EUCAST 2021, *Pseudomonas* spp. [97]	50	18	18–19
	Piperacillin-tazobactam	yes	EUCAST 2021, *Pseudomonas* spp. [97]	50	18	18–19
	Ticarcillin	yes	EUCAST 2021, *Pseudomonas* spp. [97]	50	18	
	Ticarcillin-clavulanic acid	yes	EUCAST 2021, *Pseudomonas* spp. [97]	50	18	
Carbapenems	Imipenem	yes	EUCAST 2021, *Pseudomonas* spp. [97]	50	20	
	Meropenem	yes	EUCAST 2021, *Pseudomonas* spp. [97]	24	18	
Monobactam	Aztreonam	yes	EUCAST 2021, *Pseudomonas* spp. [97]	50	18	
Cephalosporins	Cefquinome	**no**	CASFM veterinary 2013, *Enterobacteriaceae* [98]	22	19	
	Ceftiofur	**no**	CASFM veterinary 2013, *Enterobacteriaceae* [98]	21	18	
	Cefepime	yes	EUCAST 2021, *Pseudomonas* spp. [97]	50	21	
	Ceftazidime	yes	EUCAST 2021, *Pseudomonas* spp. [97]	50	17	
	Ceftolozane-tazobactam	yes	EUCAST 2021, *Pseudomonas* spp. [97]	23	23	
Phosphonic acids	Fosfomycin	yes	EUCAST 2021, *Pseudomonas* spp. Based on clinical observations [97]	12	7	
Aminoglycosides	Amikacin	yes	EUCAST 2021, *Pseudomonas* spp. [97]	15	15	
	Gentamicin	yes	EUCAST 2019, *Pseudomonas* spp. [99]	15	15	
	Netilmicin	yes	EUCAST 2019, *Pseudomonas* spp. [99]	12	12	
	Tobramycin	yes	EUCAST 2021, *Pseudomonas* spp. [97]	18	18	
Fluoroquinolones	Enrofloxacin	**no**	CASFM veterinary 2013, *Enterobacteriaceae* [98]	22	17	
	Marbofloxacin	**no**	CASFM veterinary 2013, *Enterobacteriaceae* [98]	18	15	
	Ciprofloxacin *	yes	EUCAST 2021, *Pseudomonas* spp. [97]	50	26	
	Levofloxacin	yes	EUCAST 2021, *Pseudomonas* spp. [97]	50	22	

ATU: area of technical uncertainty; CASFM: Comité de l’Antibiogramme de la Société Française de Microbiologie; DDAC: didecyldimethylammonium chloride; DS: decreased susceptibility; EUCAST: European Committee on Antimicrobial Susceptibility Testing; MDR: multidrug-resistant; R: resistance; S: susceptibility; XDR: extensively drug-resistant. *: EUCAST 2021 recommendations.
